# The Polish Panel Survey (POLPAN) dataset: Capturing the impact of socio-economic change on population health and well-being in Poland, 1988–2018

**DOI:** 10.1016/j.dib.2021.106936

**Published:** 2021-03-03

**Authors:** Olga Zelinska, Alexi Gugushvili, Grzegorz Bulczak, Irina Tomescu-Dubrow, Zbigniew Sawiński, Kazimierz M. Słomczyński

**Affiliations:** a Institute of Philosophy and Sociology, Polish Academy of Sciences, Warsaw, Poland; b Deparment of Sociology and Human Geography, University of Oslo, Postboks 1096 Blindern, Oslo 0317, Norway; c Nuffield College, University of Oxford, Oxford, UK; d CONSIRT–The Ohio State University, Polish Academy of Sciences, Columbus, OH, United States

**Keywords:** POLPAN, Health inequalities, NHP, Panel data, Post-commmunist transition, Social determinants of health

## Abstract

The Polish Panel Survey, POLPAN, one of the longest continuously run panel studies in Europe, is designed to facilitate research on the socio-economic structure, inequalities and the individual life course under conditions of social change in Poland. POLPAN is well suited for studying how women's and men's health and wellbeing are influenced by their life conditions, such as financial and social resources, that Poland's post-1989 profound socio-economic transformations impacted, and how health outcomes further shape individuals’ attitudes and behaviours. Initiated in 1987-88, POLPAN has been fielded in five-year intervals, most recently in 2018, with wave-specific samples representative of the Polish adult population and response rates for full panelists consistently above 70%. In POLPAN, health assessment measures are collected in all waves, as part of respondents’ multi-dimensional and life course inequality profile. Data on self-rated physical and psychological health, collected since 1998 (Wave Three), are complemented with respondents’ Nottingham Health Profile and core anthropometric information about personal weight and height (Wave Five onwards); health and wellbeing related reasons for work interruptions (since Wave Four); information on extensive hospital stays (Wave Six onwards) and respondents’ chronic or protracted illnesses (in Wave Six), respondents’ disability status (all waves). The newly released integrated 1988-2018 POLPAN dataset is available on Harvard Dataverse, or upon request, via e-mail: polpan@ifispan.waw.pl.

**Specifications Table**

**Table:** 

Subject	Social Sciences; Health
Specific subject area	Use of panel survey data to study causes and consequences of health outcomes and inequalities
Type of data	Primary data Table
How data were acquired	Survey For details on methodology visit http://polpan.org/en/data-and-documentation/methodology/. Documentation, in English, is available at http://polpan.org/en/data-and-documentation/documentation/.
Data format	Raw, Integrated Dataset in .sav and .csv extension
Parameters for data collection	POLPAN starts in 1988 with a large random sample of residents of Poland aged 21 to 65 years. It has been fielded every five years since, with the most recent wave in 2018. Since Wave Three, the sample of panelists is complemented with randomly drawn renewal samples of young adults. Waves maintain a representative age distribution for the country at the time of a given POLPAN survey. As a rule, respondent substitution is not allowed. Non-response follow-ups average four returns. Response rates for full panelists are consistently above 70%, but vary for intermittent panelists and the young. Data are collected, processed and stored in line with national and international regulations on data protection and privacy, including respondents’ participation consent.
Description of data collection	POLPAN data are collected by the Centre of Sociological Research at the Institute of Philosophy and Sociology, the Polish Academy of Sciences, using paper-and-pencil personal interviews (PAPI) on panelists and renewal samples of young adult residents of Poland. Quality standards are implemented and monitored throughout the survey process. The interviewer training is conducted both in Warsaw and in the 22 network units around Poland responsible for data collection; questionnaires for all waves are pretested; a minimum of 10% of interviews conducted in each wave are back-checked.
Data source location	The Institute of Philosophy and Sociology, the Polish Academy of Sciences, Warsaw, Poland
Data accessibility	Data for waves One to Four (1988-2003) of the POLPAN study are available at: Repository name: ZACAT-GESIS Data identification number: ZA4670, ZA4671, ZA4672, ZA4673, Direct URLs to complete metadata: https://dbk.gesis.org/dbksearch/sdesc2.asp?db=e&no=4670 https://dbk.gesis.org/dbksearch/sdesc2.asp?db=e&no=4671 https://dbk.gesis.org/dbksearch/sdesc2.asp?db=e&no=4672 https://dbk.gesis.org/dbksearch/sdesc2.asp?db=e&no=4673 and for waves One to Five (1988-2008) at: Repository name: Polish Social Data Archive Data identification number: P0054; P0055, P0056, P0057, P0092 Direct URL to data: http://www.ads.org.pl/wyszukiwanieE.php?typ=string&kategoria=tytop&co=polpan&send=Search POLPAN dataset is also available on Harvard Dataverse at https://dataverse.harvard.edu/dataset.xhtml?persistentId=doi:10.7910/DVN/DAPH0P. Users have to fill out the Dataset Terms. All seven POLPAN waves (separate surveys) are in the integrated .sav or .csv files. Data can be obtained upon request, via e-mail: polpan@ifispan.waw.pl.
Related research article	The POLPAN website lists over one hundred publications using POLPAN data (polpan.org/en/publications/), including health and well-being research. Below is one of the most recent research papers that uses health-related POLPAN data: A. Kiersztyn, Which Clouds Have Silver Linings? Fixed-Term Employment, Psychological Distress, and Occupational Position in Poland, International Journal of Sociology. 46 (2016) 264–287. https://www.tandfonline.com/doi/abs/10.1080/00207659.2016.1246295 This analysis is based on data from the Fifth (2008) and Sixth (2013) waves of the POLPAN. These waves can be found within the integrated POLPAN dataset on Harvard Dataverse or as separate surveys at the repositories, as specified above in the “Data accessibility” section.

**Value of the Data**

• As a panel study whose separate waves are representative of Poland's age structure at the time of the surveys, POLPAN provides both a dynamic picture of changes in the country's social structure, and allows to understand these changes under different socio-economic and political systems. Health outcomes, as a major dimension where inequality manifests, are measured in all waves, as part of respondents’ multi-dimensional and life course inequality profile.
• POLPAN panel survey can be used by sociologists, public health and social epidemiology scholars for studying how women's and men's health and wellbeing are influenced both by individual and structural characteristics that Poland's systemic transition and profound socio-economic transformations impacted, and how health outcomes further shape individuals’ social position, attitudes and behaviours.
• POLPAN's longitudinal data offers researchers the opportunity to reach deeply into the panelist's life history and search for factors determining health many years later. This enables research on causes and consequences of health outcomes and inequalities, including cohort-specific analysis.
• Starting with Wave One (1988) POLPAN collects information on respondents’ disability status. Self-rated physical and psychological health data are collected since Wave Three (1998) and are complemented, since Wave Five (2008), with respondents’ Nottingham Health Profile and core anthropometric information on individuals’ weight and height. Reasons, including illness, for work interruptions lasting three months or longer, are collected since Wave Four (2003). Wave Six (2013) asks about respondents’ chronic or protracted illnesses. Additionally, the two most recent waves (2013 and 2018) provide information on extensive hospital stays.
• POLPAN is one of the longest continuously run panel studies in Europe. Initiated in 1987-88 Poland, it has been fielded six more times, most recently in 2018. A strong foundation of social science theories informs POLPAN's operational definitions of concepts inherent to research on social stratification. Methodologically, POLPAN meets quality standards proposed in the specialized literature. All POLPAN data and their documentation are available in English, free of charge.

## 1 Data Description

### 1.1 POLPAN waves

POLPAN started in 1987-88 as a government-funded research project on social class and stratification in socialist Poland, called “Social Structure II” [1]. The study, housed at the Institute of Philosophy and Sociology, the Polish Academy of Sciences (IFiS PAN), targeted the core segment of the Polish labour force – adults aged 21 to 65 – using a cross-sectional survey design. To ensure national coverage, the project team constructed the sample using a country-wide register of households prepared in 1985-86 by the Centre for Public Opinion Research (CBOS) [2]. The survey, currently known as POLPAN's Wave One (1988), was conducted on a nationally representative sample of 5817 residents of Poland using paper-and-pencil personal interviews (PAPI). Table 1 provides the details on the fieldwork period, sampling frames and the number of completed interviews for 1988 and six subsequent POLPAN waves.

**Table 1:** POLPAN methodology, basic information.

Wave	Period of data collection	Sample	Total number of completed interviews
**First (1988)**	November 1987 - January 1988	Random sample of individuals aged 21-65 selected from a nationwide microcensus carried out by CBOS in 1986	5817
**Second (1993)**	May - December 1993	Random subsample of POLPAN 1988 respondents (aged 26-70 in 1993)	2259
**Third (1998)**	September - October 1998	Panelists interviewed in 1993 (aged 31-75), supplemented with a random sample of newly recruited respondents aged 21-30, selected from PESEL (Powszechny Elektroniczny System Ewidencji Ludności, Universal Electronic System for Registration of the Population)	2135 (1752 interviews with panelists + 383 new respondents)
**Fourth (2003)**	November 2003 to May 2004	Panelists interviewed in 1998 (aged 26-80); supplemented with a random PESEL sample of new respondents aged 21-25	1699 (1474 panelists + 225 new respondents)
**Fifth (2008)**	April - June 2008 (part one), November - December 2008 (part two)	Part one: Panelists interviewed in 2003 (aged 26-85); supplemented with a random PESEL sample of new respondents aged 21-25; Part two: oversampling of newly recruited respondents aged 21-25	1805 (1216 panelists + 589 new respondents)
**Sixth (2013)**	April - November 2013 (part one), May – December 2014 (part two)	Part one: Panelists interviewed for the last time in 1993-2008 (aged 26-90); supplemented with a random PESEL sample of new respondents aged 21-25; Part two: Panelists who participated only in 1988 survey	2780 (1699 panelists last interviewed in 1993-2008, 584 panelists participating only in 1988; 497 new respondents)
**Seventh (2018)**	April - December 2018	Panelists who were interviewed for the last time in 2013 (aged 26-95) or in 2008, if they did not participate in 2013 (aged 31-35); a random PESEL sample of new respondents aged 21-25	2161 (1875 panelists + 286 new respondents)

Soon after Social Structure II was completed, Poland embarked on the road to systemic transformation from an authoritarian political regime to a market-oriented democratic mode of governance. In the early 1990s, the study's core team decided to conduct the survey again with the same respondents to observe the dynamic aspects of the social structure, thus laying the foundation for a panel study. However, the hyperinflation of 1989–90 and the economic recession dramatically affected the budgets of academic institutions and made survey implementation a challenging task. Thanks to international financial support [3], the survey was repeated in 1993: 2500 participants were randomly selected from the 1988 sample, and 2259 were successfully interviewed.

Since 1993, POLPAN has been fielded every five years (for details on fieldwork dates, sampling frames and number of interviews, see Table 1). The core of the sample consists of panel respondents. Starting from the third wave (1998), to ensure the balance of different cohorts and age groups in the study, POLPAN adds subsamples of individuals aged 21-25. In 2013 (Wave Six), sufficient funding allowed POLPAN to recontact participants who previously had dropped out from the study, hence this wave had the larger number of panelists.

### 1.2 Health measures and main correlates

Table 2 maps the availability of health assessment measures in POLPAN. Questions on self-rated physical and mental health appear since 1998. All respondents are asked, using 4-point scales, “How do you evaluate the state of your physical health in comparison with the state of physical health of other (the majority) people your age?” and “How would you evaluate your psychological state, your mood?”

**Table 2:** Health assessment measures in POLPAN.

POLPAN Wave	Age category	Self-assessed physical health	Self-assessed mental health	Height and weight	Living with chronic illness	Hospital stays in past five years	Health-related reasons for interrupting work	Information on disability	NHP Part one	NHP Part two
First (1988)	All	No	No	No	No	No	No	Yes	No	No
Second (1993)	All	No	No	No	No	No	No	Yes	No	No
Third (1998)	All	Yes	Yes	No	No	No	No	Yes	No	No
Fourth (2003)	All	Yes	Yes	No	No	No	Yes	Yes	No	No
Fifth (2008)	All	Yes	Yes	Yes	No	No	Yes	Yes	Yes, 38 items	Yes, 7 items
Sixth (2013)	21-30	Yes	Yes	Yes	Yes	Yes	Yes	Yes	No	Yes, 4 items
	31-70	Yes	Yes	Yes	Yes	Yes	Yes	Yes	Yes, 38 items	Yes, 7 items
	71-90	Yes	Yes	Yes	Yes	Yes	No	Yes	Yes, 38 items	Yes, 7 items
Seventh (2018)	21-50	Yes	Yes	Yes	No	Yes	Yes	Yes	Yes, 19 items	No
	51-70	Yes	Yes	Yes	No	Yes	Yes	Yes	Yes, 38 items	Yes, 7 items
	71-95	Yes	Yes	Yes	No	Yes	No	Yes	Yes, 38 items	Yes, 7 items

Starting with Wave Five (2008), POLPAN adds questions of the Nottingham Health Profile (NHP) [4], [5], [6]. Developed in the UK as a population survey tool, NHP is considered a valid and reliable indicator of individuals’ health status [4], is easily administered, contains unambiguous response categories, and consists of two parts. Part one of the profile contains 38 questions, grouped into six areas of health and wellbeing referring to physical mobility (eight items), pain (eight items), sleep (five items), social relationships (five items), emotional life (nine items) and energy level (three items). The statements are presented in random order to avoid contamination of assessment. Part two of the profile contains information on seven areas of life affected by health: employment, work around the house, social life, personal relationships, sex life, hobbies and holidays [5]. For the analysis of NHP, responses are weighed, summed up, and a total score for each area is calculated. The score stands for “perceived dysfunction in different domains” [5].

In 2008, the original 38-category test was administered to all respondents. Since then, POLPAN selectively uses parts one and two of the NHP, depending on interviewees’ age (see Table 2, columns 6-7). This decision was informed by analyses of the 2008 data showing little variability in health problems among younger respondents.

From Wave 2008 onward, all POLPAN respondents are asked to provide information about their height (in centimetres) and weight (in kilograms). Researchers can thus calculate the body mass index and investigate its contemporary and life course predictors. Since 2003, POLPAN collects information on health and wellbeing-related work interruptions of at least three months, including maternity leaves, and illness or poor health [7].

Respondents’ disability status, an important health indicator, can be traced already in POLPAN Wave One (1988). In all POPLAN waves disability pension being part of their answers about sources of income. In Waves Four (2003), Five (2008), Six (2013) and Seven (2018) disability is among the reasons for work interruptions (see above). In 2003 and 2018 respondents were also directly asked about their disability status, when it occured, and whether they required special care.

POLPAN's Sixth Wave (2013) asked respondents if they suffered chronic or protracted illnesses, such as asthma, allergy, circulatory system ailments, cancer, diabetes or depression (yes-no questions). Additionally, the most recent two waves (2013 and 2018) collect retrospective data on extended hospital stays. All respondents are asked whether, in the last five years, they were hospitalised for longer than seven days.

In POLPAN, health assessment indicators are part of the study's set of measures intended for analyses of social inequality across multiple dimensions. All seven POLPAN waves provide detailed socio-demographic information on respondents (e.g. gender age, education), their family life (e.g. marital status, having children) and family history (e.g. father's education and social position). Work and life situation indicators, such as respondents’ employment and occupational history, income data at both the individual and household levels, and information on the composition of the household, living conditions and possession of durable goods, are collected in each POLPAN wave and for all age groups. Information about time spent on house chores is available selectively. Respondents’ social capital can be evaluated based on the questions related to the number of friends, organisational membership and religious affiliation. Thanks to POLPAN's longitudinal structure, researchers can “track” causes of respondents’ good or bad health using indicators available in waves that precede the collection of health measures. At the same time, they can examine the consequences of health inequalities, *ceteris paribus*. POLPAN's questionnaires, available also in English, provide the study's full survey item coverage [8].^1^

## 2 Experimental Design, Materials and Methods

### 2.1 Sampling and weights

Wave-specific POLPAN samples can be considered representative of the Polish population aged 21 and older at the time of the survey. The upper limit of respondents’ age depends on the wave and has increased progressively, from 65 in 1988, to 95 in 2018. Given oversampling of young, cross-sectional analyses of data from Waves Five (2008) through Seven (2018) data require the use of weight variables for the shares of various age groups. Post-stratification weights for 2008, 2013 and 2018 are available in POLPAN dataset.

### 2.2 Data quality

Data quality is a major consideration in POLPAN. The study considers recommendations from the specialised literature on minimizing errors in representation and measurement [11], [12], [13], and maximizing the study's responsiveness to users’ needs [14]. To strengthen representation, POLPAN relies on random sampling when appropriate (e.g. all renewal samples), and non-response follow-ups (on average, four returns). Beyond the first two waves, respondent substitution is not permitted. Response rates (RR) for full panelists (i.e. participants in all waves) are consistently above 70%, but vary for intermittent panelists (i.e. participants in at least two but not all seven POLPAN waves) and the young. For example, in 2018 RR for the young was 54%, while in 2013 it was 69% [10].

Continuity in POLPAN's research team, and the strong collaboration with the data collection organisation, the Centre of Sociological Research (ORBS) at IFiS PAN, ensure that quality standards are implemented and monitored throughout the survey process. For example, interviewer training is conducted both in Warsaw and in the 22 network units around Poland responsible for data collection; questionnaires for all waves are pretested; a minimum of 10% of interviews conducted in each wave are back-checked [15].

### 2.3 Funding

POLPAN waves are funded separately. Funding for 2018 and 2013 came from the Polish National Science Centre. Past funding sources highlight the extent of international involvement and include not only Polish institutions – State Committee for Scientific Research, Ministry of Science and Higher Education, Central Fund for Research and Development, and Polish Academy of Sciences (PAN) – but also United States Information Agency, The Ohio State University (OSU), US National Council for Eurasian and East European Research, and the Norwegian Research Council, and IREX. The project receives organisational support from IFiS PAN and CONSIRT - Cross-National Studies: Research and Training Program at OSU and PAN [16].

## Ethics Statement

POLPAN data are collected, processed and stored in line with national and international regulations on data protection and privacy, e.g. General Data Protection Regulation (GDPR, EU-Lex Document 32016R0679). This includes securing all participants’ informed consent. All publicly available information on POLPAN participants is anonymised.

## Declaration of Competing Interest

The authors declare that they have no known competing financial interests or personal relationships which have or could be perceived to have influenced the work reported in this article.

## Supplementary materials

Image, application 1

## Footnotes

1 All POLPAN questionnaires feature a common, stable core of questions asked to all participants. At the same time, there is some variation in questionnaire content, within and between survey waves. First, to account for differences in participation status among POLPAN respondents (i.e. panelists, intermittent panelists and new recruits), questions that specifically target new recruits, and respondents who skipped waves are included. Thus, within-wave variation in questionnaire content appears. Next, in light with the study's theoretical and methodological needs, certain quetions were added or dropped. This is the source for between-wave variation in questionnaires. Information about wave and questionnaire version is preserved in POLPAN variable names.
