# Supplementary material for: The Polish Panel Survey (POLPAN) dataset: Capturing the impact of socio-economic change on population health and well-being in Poland, 1988–2018
Source: Data Brief. 2021 Mar 3;35:106936. doi: 10.1016/j.dib.2021.106936 (PMC7988276; doi:10.1016/j.dib.2021.106936)
Supplement: Supplementary file 1 [file mmc1.docx]

Structure of the integrated dataset: Brief Overview

Block 1: Variables constructed by Data providers

In POLPAN 1988-2018, the first block of variables, starting with LICENSE (row 1), and ending with EDUC2018 (row 107) is created by data providers. Within this block, variables in rows 1-59 facilitate data management and selection of appropriate waves and subsets of respondents, including weighting.*

Variables in rows 60-107 provide common values for the measures of respondents’ occupational careers and education across POLPAN’s seven waves. These harmonized indicators are available in addition to the original, wave-specific, measures of respondents’ occupation and education that informed their construction.

Block 2: Variables of individual POLPAN waves

The second block of variables in the integrated POLPAN 1988-2018 file provides the information collected in a given POLPAN wave. The data are stacked in chronological order, starting with wave 1988. The variables START1988, START1993, START1998, START2003, START2008, START2013, and START2018 mark the beginning of within-wave variables.

Within each wave, all variables start with the same letter, as follows:

Z for 1988 variables

Y for 1993 variables

X for 1998 variables

W for 2003 variables

V for 2008 variables

U for 2013 variables

T for 2018 variables

* The weight variables WGT2008, WGT2013 and WGT2018, respectively, facilitate cross-sectional analyses on the 2008, 2013 or 2018 POLPAN data that intend to reproduce the actual structure of the population of people aged 21 years or older.
